# An Observational Study of the Role of Adiponectin and Vitamin D in Pediatric Asthma and Obesity

**DOI:** 10.3390/children13040514

**Published:** 2026-04-07

**Authors:** Jelena Knežević, Olga Malev, Marcel Lipej, Ivana Banić, Mirjana Turkalj

**Affiliations:** 1 Product Development Project Management Office, Genera Inc. (Part of Dechra Pharmaceuticals PLC Group), Svetonedeljska cesta 2, 10436 Kalinovica, Croatia; 2 Division for Materials Chemistry, The Ruđer Bošković Institute, 10000 Zagreb, Croatia; omalev@irb.hr; 3 IT Department, Srebrnjak Children’s Hospital, 10000 Zagreb, Croatia; 4 Department of Medical Research, Srebrnjak Children’s Hospital, 10000 Zagreb, Croatia; 5 Department of Allergy, Clinical Immunology and Rheumatology, Srebrnjak Children’s Hospital, 10000 Zagreb, Croatia; mturkalj@bolnica-srebrnjak.hr; 6 School of Medicine, Catholic University of Croatia, 10000 Zagreb, Croatia; 7 Faculty of Medicine, J. J. Strossmayer University of Osijek, 31000 Osijek, Croatia

**Keywords:** asthma, obesity, adiponectin, vitamin D, lung function

## Abstract

Background/Objectives: The co-occurrence of asthma and obesity presents a significant clinical challenge, but the underlying mechanisms remain unclear. Reduced adiponectin and vitamin D levels have been associated with both conditions, suggesting that their potential modulatory roles warrant further investigation. This study aimed to evaluate whether vitamin D and adiponectin levels differ among pediatric groups defined by their asthma and obesity status, to better characterize the metabolic and inflammatory profile of the obesityasthma phenotype. Methods: A total of 120 participants aged 6–18 were enrolled and categorized into four groups: Asthma (*n* = 30), Obesity (*n* = 30), Asthma + Obesity (*n* = 30), and Control group (*n* = 30). All participants underwent lung function testing, anthropometric assessment and measurement of fraction of exhaled nitric oxide (FeNO). Participants were further categorized according to BMI percentiles. Adiponectin levels were measured using ELISA, while vitamin D levels were detected using HPLC. Results: Vitamin D levels and lung function parameters did not differ across groups, although deficiency was most prevalent in the obesity group. FeNO was elevated in asthmatics relative to obese children (*p* = 0.038) and in obese asthmatics compared with both controls (*p* = 0.040) and obese children (*p* = 0.021). Adiponectin levels were lower in obese asthmatic children compared to the controls (*p* = 0.024). A similar difference was observed between the controls and obese asthmatics among children with low vitamin D levels (*p* = 0.014). Conclusions: The dominant mechanisms underlying the obesity–asthma phenotype remain unclear; however, our findings indicate a link between adiponectin dysregulation and heightened airway inflammation, as evidenced by increased FeNO levels, though the precise pathways involved are still not well-understood. The role of vitamin D appears less consistent. These results highlight the need for further research to clarify the interplay between metabolic and inflammatory pathways and to support more personalized management strategies in children with obesity-related asthma.

## 1. Introduction

Asthma and obesity are highly prevalent chronic disorders in pediatric and adolescent populations, and their co-occurrence presents a unique clinical challenge. Due to overlapping inflammatory pathways, obesity-related asthma is now recognized as a distinct phenotype, often characterized by poorer disease control and reduced treatment responsiveness. Beyond systemic inflammation, the relationship between obesity and asthma is further complicated by the patient’s atopic status and the age of onset. Two major obesity–asthma subphenotypes have been proposed: early-onset asthma, which is characterized by Th2-driven inflammation, atopy, and allergen sensitization, and is subsequently complicated by obesity; and late-onset asthma, which is often non-atopic and considered primarily obesity-driven [1]. In addition to obesity being an established independent risk factor for asthma, certain studies also suggest that asthma itself may contribute to the development of obesity, particularly in children with severe allergic asthma, possibly due to reduced physical activity or corticosteroid treatment [2,3].

Obesity may exacerbate asthma symptoms and severity through mechanisms that are not yet fully understood [4,5,6]. One prominent factor linking these conditions is adipose tissue, which in obese individuals produces altered levels of adipokines and pro-inflammatory cytokines, contributing to systemic inflammation. In obesity, increased levels of adipokines such as leptin and resistin are observed, along with elevated concentrations of pro-inflammatory cytokines, including tumor necrosis factor (TNF α) and nuclear factor NF-kB, while adiponectin levels are significantly reduced [7,8,9]. This obesity-induced systemic inflammation, mediated by cytokines and adipokines, is hypothesized to play a crucial role in the pathogenesis of asthma in obese children, potentially leading to enhanced airway inflammation and increased risk of exacerbations [10,11,12,13,14].

Adiponectin, a 244-amino acid protein with a molecular weight of 28 kDa, is a biologically active hormone [7,15] with well-established anti-inflammatory properties. It counteracts cytokines such as IL-6 and TNF α, reduces NF-kB activity, and upregulates the expression of anti-inflammatory mediators, including IL-10 and IL-1 receptor antagonists [7,8,9]. This inflammatory state may significantly influence the risk of developing asthma, as well as its phenotype and prognosis, particularly through effects on Th1/Th2 balance, lung development, airway smooth muscle function, and overall airway responsiveness [16]. Despite epidemiological studies consistently reporting reduced adiponectin levels in obese children with asthma, the specific associations between adiponectin levels and asthma symptoms or lung function remain debated. Studies report negative associations, while others describe positive correlations [16,17,18,19,20,21]. These discrepancies may reflect differences in study design, population characteristics, or other confounding factors. Nevertheless, evidence from both animal and human studies suggests that lower adiponectin may contribute to increased asthma severity in obese children [19,22,23].

Vitamin D is another modulator gaining attention for its role in obesity and asthma. Vitamin D has been extensively studied across different regions, populations, and ethnic groups. However, considerable debate remains regarding its optimal concentration and reference values, particularly in the pediatric population [24,25,26,27,28]. Several observational studies have reported vitamin D deficiency even in healthy children, highlighting the potentially high global prevalence of insufficient vitamin D levels. These levels are influenced not only by dietary habits and sun exposure, but also by age, sex, and socioeconomic status [24,25,28,29,30,31,32,33,34]. Furthermore, both the prevalence and severity of vitamin D deficiency are strongly affected by geographic location and seasonal UV exposure. This study was conducted in Croatia, situated between approximately 42° N and 46° N latitude, well above the 35-degree latitude threshold. At these latitudes, cutaneous vitamin D3 synthesis from UVB radiation is limited or absent during winter months, contributing to a high prevalence of deficiency in the population. This geographic context is an important factor in interpreting the seasonal variation in vitamin D levels observed in Central European pediatric cohorts [35].

Low vitamin D levels have been reported in both asthma and obesity, placing obese asthmatics at an even higher risk of deficiency and more severe asthmatic symptoms [14]. In addition, vitamin D may exert a modulatory effect on the expression of genes involved in the synthesis and secretion of adipokines. This is supported by previous studies reporting significantly lower adiponectin levels in obese children with vitamin D deficiency [36,37]. Furthermore, some studies suggest an inverse relationship between asthma severity and serum vitamin D levels, with vitamin D supplementation showing potential benefits in improving adiponectin concentrations and asthma control [37,38]. However, the evidence supporting vitamin D supplementation as an effective intervention for improving adiponectin levels and asthma outcomes in obesity remains inconclusive, warranting further investigation.

While research continues to explore the link between asthma and obesity in children, relatively few studies have specifically examined the roles of adiponectin and vitamin D in the context of inflammation. Therefore, this observational study aimed to evaluate whether vitamin D and adiponectin levels differ among pediatric groups defined by their asthma and obesity status, to better characterize the metabolic and inflammatory profile of the obese-asthma phenotype. By analyzing these biomarkers, the study sought to improve the understanding of how obesity may influence asthma exacerbations, ultimately contributing to enhanced management strategies for this vulnerable pediatric population.

## 2. Materials and Methods

### 2.1. Participant Selection

A cohort of 120 participants (aged 6–18 years) from a homogenous population (Caucasian children in Croatia) was recruited to this observational case–control study at the Srebrnjak Children’s Hospital (SCH), Zagreb, Croatia outpatient clinic. Informed consent was obtained from the children’s parents/legal guardians. All children and adolescents were enrolled in the study after signing the informed consent, according to local legislation. The study protocol was approved by the local Ethics Committee (Supplementary Materials). Inclusion criteria for participants with asthma were an established diagnosis of asthma for at least one year in accordance with the GINA 2023 guidelines and the ATS/ERS recommendations [39,40]. For the healthy control group, the absence of asthma was confirmed by a negative clinical history of respiratory symptoms and normal baseline spirometry without variable airflow limitations. The inclusion and exclusion criteria were assessed and defined by an allergy specialist with over 20 years of experience in the field. Body mass index (BMI) values obtained for each subject were compared with percentile growth curves specific to the child’s gender and age. Inclusion criteria for overweight and obesity were BMI values equal to or greater than the 85th percentile but less than the 95th percentile (overweight with an increased risk for obesity and related comorbidities) and BMI values equal to or greater than the 95th percentile, specific for age and sex (obesity). Exclusion criteria for all participants were: immunodeficiency; malignancies; autoimmune diseases; diagnosis of diabetes type 1 or diabetes mellitus; diagnosis of cystic fibrosis; primary ciliary dyskinesia; hematologic disorders; vitamin D supplementation in the last 3 months; therapy that could influence glucose levels; known inborn or perinatal pulmonary disease; pulmonary malformation; oxygen therapy after birth with a duration of more than 24 h; ventilator support or mechanical ventilation after birth; heart failure diagnosed after birth affecting pulmonary circulation; major respiratory diseases such as interstitial lung disease, recent asthma-related visit to emergency department (in the past three weeks), and recent anaphylactic reaction to an allergen (in the past month). Moreover, all participants were excluded from study visits and biomaterial collection in the case of fever of at least 38.5 °C during the last two weeks prior to the planned visit.

### 2.2. Lung Function

Lung function and exhaled nitric oxide test (FeNO) measurements were performed for all participants to assess airway inflammation and eosinophilic inflammation in the lower airways. Spirometry was conducted using a computerized pneumotach (GANSHORN Medizin Electronic GmbH, Niederlauder, Germany) following ATS guidelines [41,42]. Forced Expiratory Volume in 1 s (FEV1) and Forced Vital Capacity (FVC) were recorded and expressed as the % of predicted values, while FEV1/FVC ratio was reported as an absolute value [43]. Values of FVC (>80% of predicted or above the lower limit of normal), FEV1 (>80% of predicted or above the lower limit of normal), and FEV1/FVC ratio (FEV1/FVC) (>0.90) were interpreted as normal. The obstructive pattern was defined by decreased FEV1 and FVC (<80% of predicted or below the lower limit of normal) and decreased FEV1/FVC. Percentage of FEV1 was used to predict the severity of airway obstruction in children: FEV1 > 80% as an indication of mild obstruction; FEV1 60–80% as an indication of moderate obstruction; FEV1 < 60% as an indication of severe obstruction [39,43,44,45]. In accordance with ATS standards, FeNO measurements were strictly performed before any spirometry tests, as forced exhalations can transiently reduce exhaled nitric oxide levels. FeNO levels were measured with the single exhalation method at 50 mL/s for 10 s using a NiOX Analyzer (Aerocrine AB, Solna, Sweden) in accordance with the ERS/ATS recommendations [40,46].

Inhaled corticosteroid (ICS) treatment was included as a clinical variable. Due to high variability in class and dose, ICS exposure was recorded only as a binary variable (receiving ICS treatment: yes/no).

### 2.3. Vitamin D Determination

Whole blood samples were collected in anticoagulant-treated tubes (EDTA) and centrifuged at 3500 rpm for 10 min at 4 °C to isolate plasma. Plasma concentration of 25-hydroxyvitamin D2 and 25-hydroxyvitamin D3 (25-OH D2 and 25-OH D3) were quantified by High Performance Liquid Chromatography (HPLC). Levels of 25(OH)D were measured using commercially available diagnostic kit (Vitamin 25-OH-D3 and Vitamin 25-OH-D2 in plasma by UV–FAST, Eureka Lab Division, Chiaravalle, AN, Italy). Lyophilized serum controls and calibrators provided with the kit were reconstituted in HPLC grade water according to the manufacturer’s instructions, aliquoted, and stored at −20 °C. After deproteinization, prepared controls, calibrators, and plasma samples were loaded onto a Clean-up Column, processed, eluted and collected in a glass tube. Chromatographic analysis was conducted on an 1260 Bio-Inert HPLC system (Agilent Technologies, Santa Clara, CA, USA) equipped with a UV detector. Selected analytes were separated on a C18 Reversed-Phase column (POROSHELL EC 4.6 × 50 mm, 2.7 µm, Agilent Technologies), with a prefilter (Javelin, Fisher Scientific, Pittsburgh, PA, USA) at a flow rate of 0.8 mL/min and a temperature of 20 °C. Injection volume was 50 μL, and detection was performed at a wavelength of 265 nm. Data acquisition and analyses were conducted using OpenLab ChemStation software version C.01.10 (Agilent Technologies, Santa Clara, CA, USA). While both vitamin D2 and vitamin D3 were measured, only serum vitamin D3 concentrations were reported due to their greater biological and clinical relevance. This approach is consistent with common clinical and nutritional practice, as vitamin D3 is significantly more effective than vitamin D2 at raising and sustaining total serum 25(OH)D levels. Levels of 25-OH vitamin D3 were expressed in µg/L. Values equal to or greater than 20 µg/L were considered normal, values between 10 and 19.99 µg/L were defined as insufficiency, and values lower than 10 µg/L were defined as deficiency. Values below 10 µg/L were defined as serious deficiency [24,47,48,49,50,51,52,53,54].

### 2.4. Adiponectin Determination

Whole blood samples were collected in anticoagulant-treated tubes (EDTA), followed by centrifugation at 3000× *g* for 10 min, at room temperature. Adiponectin serum levels were measured using a commercially available Enzyme Linked Immunosorbent Assay (ELISA) kit (Human Adiponectin ELISA kit, Invitrogen, Carlsbad, CA, USA). Samples, previously stored at −80 °C, were thawed at room temperature and diluted 1:5000 (according to manufacturers’ protocol). Diluted samples were transferred onto a 96-well plate, incubated with Biotin-Conjugate for 2 h, then washed and incubated with Streptavidin-HRP for one hour at room temperature. Following additional washing steps, TMB Substrate Solution was added and incubated for 30 min, after which the reaction was stopped with the Stop Solution. Absorbance was immediately measured at 450 nm. The standard curve was created by plotting the mean absorbance for each standard concentration on the ordinate against the adiponectin concentration on the abscissa. Concentration of circulating human adiponectin for each sample was calculated using a standard curve according to the manufacturers’ recommendations.

### 2.5. Statistical Analysis

The distribution of continuous variables was assessed using the Kolmogorov–Smirnov test. Comparisons between groups for continuous variables were performed using one-way ANOVA for normally distributed data or the Mann–Whitney U test for non-normally distributed data. Differences between categorical variables were calculated using the χ^2^ test. Continuous variables are presented as median values and interquartile range (IQR) for non-normally distributed data or mean and standard deviation (SD) for data with normal distribution. Categorical data are expressed as numbers (N) and percentages (%).

The data were analyzed using GraphPad 8.0. The threshold value of *p* < 0.05 was considered statistically significant for all performed tests.

## 3. Results

### 3.1. Demographic Characteristics and Adiposity Measurements

The study included four groups of children (30 children per group): controls, children with obesity, children with asthma, and children with both obesity and asthma. The average age and male/female ratio were comparable between the groups (Table 1). BMI values, calculated using sex-specific BMI-for-age percentiles, are displayed in Table 1.

Table 2 displays differences in ICS use between asthmatic patients with optimal weight and those with obesity, showing that ICS therapy was significantly less frequent in obese asthmatics compared to asthmatics with optimal weight (30% vs. 70%, *p* = 0.002).

### 3.2. Spirometry Measurements

**Figure 1:**
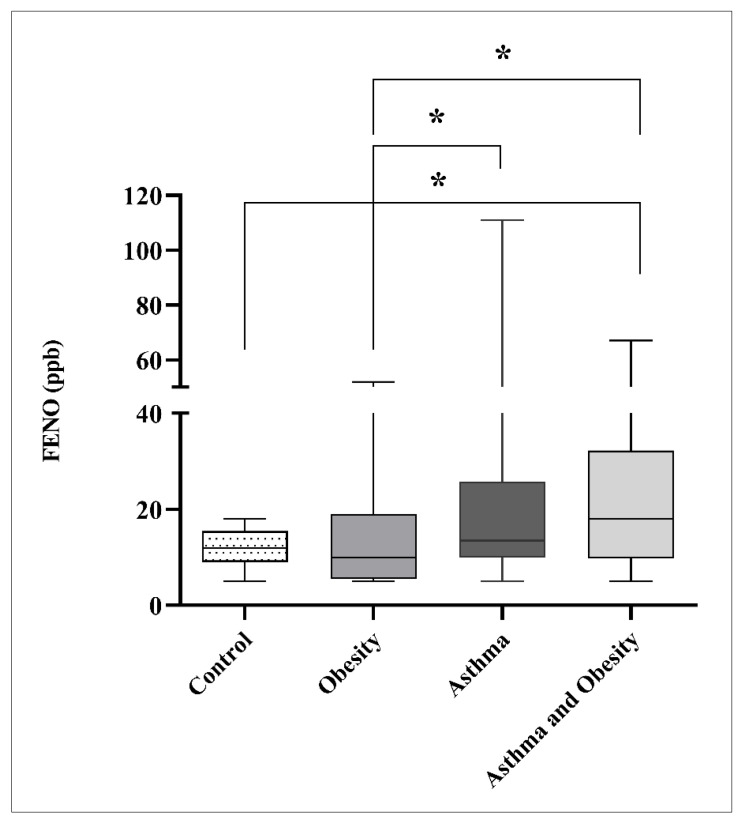
Comparison of median FeNO levels between groups. The box plots represent the median (horizontal line) and interquartile range (IQR, 25th–75th percentiles); whiskers indicate the minimum and maximum values. Differences were analyzed using the Mann–Whitney U test. *p* < 0.05 denotes statistical significance(* *p* < 0.05).

Table 3 displays the results of spirometry, expressed as a percentage of the predicted value adjusted for age, gender, weight and height. Spirometry parameters, including FEV1, FVC, and the FEV1/FVC ratio, were within normal ranges across the groups, with no indication of lung function impairment at the time of measurement. No statistically significant differences in FEV1, FVC, or FEV1/FVC ratio were observed among the groups. However, FeNO levels were higher in asthmatic (13.5 [10.0–25.8] ppb; *p* = 0.038) and obese asthmatic children (18.0 [9.8–32.3] ppb; *p* = 0.021) than in obese children (10.0 [5.5–19.0] ppb) and was higher in obese asthmatics compared with the controls (12.0 [9.0–15.5]; (*p* = 0.040) (Figure 1, Table A1). Comparing the results measured only for children with low vitamin D levels, no differences in measured FeNO values were detected between the groups. Here, the highest FeNO values were measured in obese asthmatics, but did not reach statistical significance when compared to other groups.

### 3.3. Vitamin D Status

The distribution of 25-OH vitamin D3 status across the study groups is summarized in Table 4. Overall, low vitamin D levels (<20 µg/L) were observed in 40% of controls, 53% of children with obesity, 43% of those with asthma, and 40% of children with both asthma and obesity. Vitamin D deficiency (<10 µg/L) was most frequent in the obesity group, while the prevalence of sufficient vitamin D levels (≥20 µg/L) ranged from 47% to 60% across the groups.

**Figure 2:**
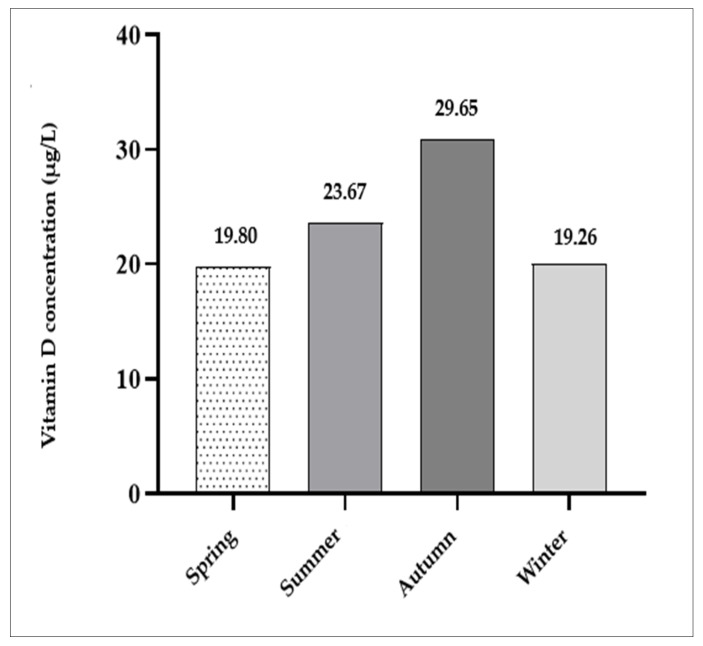
25-OH vitamin D3 levels in relation to the season of the year.

In addition, seasonal variation was noted, with higher mean 25-OH vitamin D3 levels in summer (23.7 µg/L) and autumn (29.7 µg/L) compared to spring (19.8 µg/L) and winter (19.3 µg/L) (Figure 2). Also, the highest number of children with 25-OH vitamin D3 deficiency was recorded in the first and last part of the year compared to mid-year months and longer exposures to the sun.

### 3.4. Adiponectin Levels

**Figure 3:**
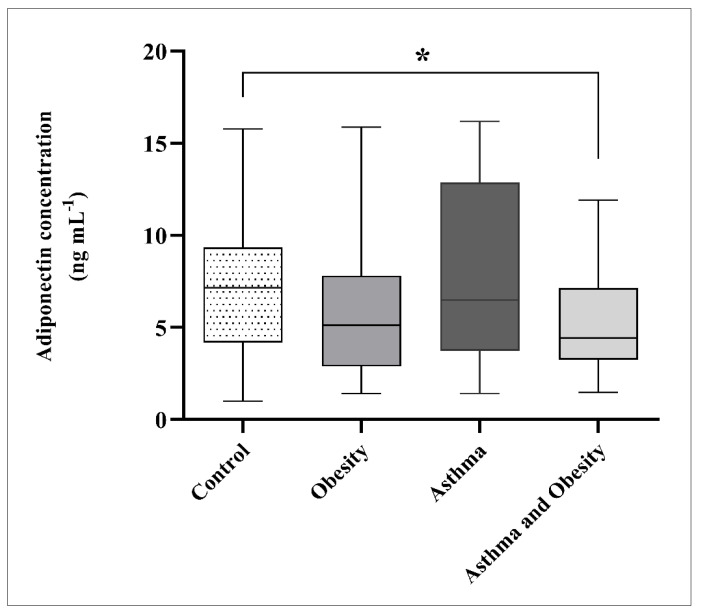
Comparison of median adiponectin levels between groups. The box plots represent the median (horizontal line) and interquartile range (IQR, 25th–75th percentiles); whiskers indicate the minimum and maximum values. Differences were analyzed using the Mann–Whitney U test. *p* < 0.05 denotes statistical significance (* *p* < 0.05).

Adiponectin levels were reduced in obese children and obese asthmatics relative to the controls and non-obese asthmatic children. A difference was observed between obese asthmatics and controls (*p* = 0.024) (Figure 3), whereas comparisons between controls and obese children (*p* = 0.100) and between asthmatics and obese asthmatics (*p* = 0.065) were not significant (Table A2). No difference was detected between the control and asthma groups.

In children with low 25-OH vitamin D3 levels, adiponectin concentrations were higher in obese asthmatics than in the controls (*p* = 0.014). A similar trend was observed between obese asthmatics and asthmatics, without reaching significance (*p* = 0.066) (Table 5).

**Figure A1:**
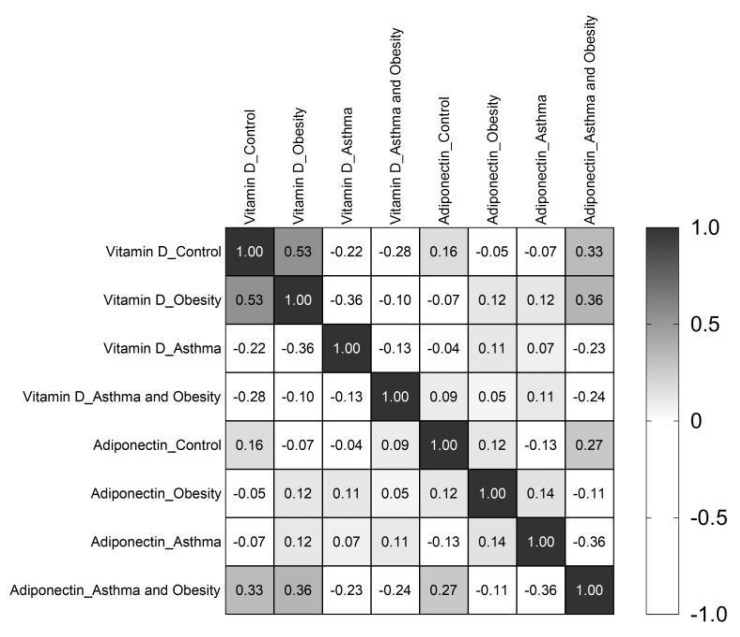
Correlation analysis between 25-OH vitamin D3 and adiponectin across the groups.

Additionally, Spearman correlation coefficients were calculated to assess the relationship between serum 25-OH vitamin D3 and adiponectin for the whole cohort and by groups. No significant correlation was observed overall. While a weak association was noted specifically in the obese asthmatic group, it did not reach statistical significance (Figure A1), suggesting the absence of a robust relationship.

## 4. Discussion

The findings of this study provide insight into the roles of adiponectin and vitamin D in the mechanisms underlying obesity-related asthma (ORA). However, these results should be interpreted within the context of the heterogeneous nature of ORA, which involves numerous immunological, metabolic, and mechanical pathways rather than a single inflammatory mechanism [54,55,56,57].

Elevated FeNO levels observed in both normal-weight and obese asthmatic children, with a more pronounced increase in the latter, suggest a mixed inflammatory phenotype in which obesity-related systemic inflammation coexists with eosinophilic airway inflammation. FeNO is a well-established marker of eosinophilic airway inflammation, reflecting increased activity of inducible nitric oxide synthase in airway epithelial cells. Therefore, elevated FeNO levels in these participants indicate ongoing airway inflammation even in the absence of measurable airflow limitation, as highlighted in the pediatric GINA guidelines [39]. In obese asthmatics, this finding may reflect the combined effects of asthma-related airway inflammation and obesity-associated systemic inflammation.

The observation of elevated FeNO levels in the obese-asthmatic group contributes to the ongoing debate regarding the inflammatory phenotype of ORA. While traditional models characterize ORA as a predominantly non-T2, neutrophilic phenotype associated with low FeNO and Th1/Th17 dominance [54,56], our findings suggest greater heterogeneity, with contributions from both T2 and non-T2 inflammatory pathways. In this context, the presence of obesity-driven systemic inflammation alongside eosinophilic airway inflammation supports the concept of a mixed inflammatory phenotype. The pathogenetic pathway for this phenotype likely involves chronic low-grade systemic inflammation originating in expanded adipose tissue.

Adipose tissue functions as an active endocrine organ, secreting pro-inflammatory cytokines, such as IL-6 and TNF-α, as well as adipokines that may enhance inflammatory pathways involved in asthma pathophysiology. This interpretation is consistent with previous studies suggesting that BMI influences airway inflammation, although its specific impact on FeNO remains inconsistent due to variations in adiposity measures [58,59,60]. Importantly, elevated FeNO levels have been reported in patients with normal spirometric parameters, indicating that normal lung function does not necessarily reflect the absence of airway inflammation or optimal asthma control [39,61,62,63]. Da Silva Salviano et al. further highlighted FeNO measurement as a potential non-invasive method for the early detection of airway inflammation, potentially preceding clinical symptoms or spirometric abnormalities [63]. Accordingly, the elevated FeNO values observed in obese asthmatics may suggest a more complex inflammatory phenotype, although it remains uncertain whether this inflammation is primarily driven by obesity, asthma, or their interaction.

A key limitation of this study is the potential confounding effect of ICS treatment on FeNO levels. ICS therapy suppresses airway inflammation and reduces FeNO, making it a critical determinant of this biomarker. The observed imbalance in ICS use between groups, with higher usage among non-obese asthmatic children provides a plausible alternative explanation for the elevated FeNO observed in obese asthmatics. Reduced ICS use in this subgroup may result in decreased suppression of airway inflammation, leading to higher FeNO levels independent of obesity-specific mechanisms. Furthermore, the lack of data on asthma control and treatment adherence limits the ability to determine whether elevated FeNO reflects inadequate treatment, poorer disease control, or true pathophysiological differences [39]. Therefore, conclusions regarding the obesity-driven inflammatory pathway should be interpreted with caution. Future studies incorporating detailed information on ICS dosage, adherence, and validated measures of asthma control are needed to better differentiate treatment effects from disease-specific mechanisms.

In addition to increased FeNO levels, obese asthmatics in our cohort exhibited significantly lower plasma adiponectin levels, consistent with previous studies reporting reduced adiponectin concentrations in both obese individuals and patients with obesity-associated asthma [8,11,12,15,64]. Our findings, in agreement with Azuma et al. [64], suggest that decreased adiponectin levels may be associated with increased eosinophilic inflammation in this population.

The coexistence of lower adiponectin levels and higher FeNO values observed in our study supports the hypothesis that obesity-related metabolic dysregulation may enhance inflammatory pathways involved in asthma. Further analysis demonstrated that obese asthmatic children with insufficient 25-OH vitamin D3 levels had lower adiponectin concentrations compared with those with optimal 25-OH vitamin D3 status. This is consistent with previous evidence indicating that 25-OH vitamin D3 deficiency may impair adipose tissue endocrine function, leading to reduced adiponectin secretion [13,65,66]. In line with this, Rashidmayvan et al. reported a positive association between vitamin D status and adiponectin levels and further showed that vitamin D supplementation may increase the adiponectin concentrations, suggesting a potential anti-inflammatory effect [67]. These findings support the rationale for future prospective trials investigating the effect of vitamin D supplementation on adiponectin levels, especially in Croatia, where an increase in obesity and asthma is recorded every year.

Obesity has also been strongly associated with vitamin D insufficiency, particularly in asthmatics, assessing its association to increased asthma symptoms. The higher frequency of vitamin D deficiency observed in obese asthmatic children supports the hypothesis of vitamin D sequestration in adipose tissue, which may reduce its bioavailability and anti-inflammatory effects.

Although there is evidence supporting this, many studies have also reported decreased vitamin D levels among healthy children worldwide, with conflicting results regarding the need for vitamin D supplementation [24,25,30,31,32,33,34]. Recent systematic reviews and meta-analyses have revealed variable associations between vitamin D status and inflammatory biomarkers, including IgE, eosinophils, and FeNO [68,69]. Interventional studies further suggest that vitamin D supplementation does not consistently improve lung function or reduce FeNO levels, although it may decrease asthma exacerbation in certain subgroups [70,71]. Moreover, seasonal variation likely influenced 25-OH vitamin D3 levels in this study, as samples were collected throughout the year without strict seasonal control. Levels were generally lower in winter and spring and higher in summer and autumn, reflecting the geographic location of the study site. Croatia lies at approximately 42° N to 46° N latitude, where UVB radiation is insufficient for vitamin D production, especially during winter months, roughly between November and February or March. This seasonal variability may limit generalizability but is representative of populations in similar latitudes, where “vitamin D winters” significantly contribute to deficiency [35]. Additionally, a recent study by Kelemen et al. suggested that up to 90% of Croatian individuals may have suboptimal vitamin D levels, with no significant association with sex or age, highlighting the need for preventive strategies and health education on a national level [72].

No significant differences in FEV1 or FVC were observed, consistent with previous findings showing overlapping forced spirometric values in obese asthmatic and non-asthmatic children compared to their peers with normal body weight [73]. The discrepancies between normal lung function and elevated inflammatory biomarkers further support evidence that airway inflammation may be present even in the absence of detectable airflow limitation [54,74]. Although the relationship between obesity, adiponectin, FeNO, and vitamin D have previously been described, this study is novel in its simultaneous evaluation of these factors in a pediatric population. This integrated approach provides a more comprehensive understanding of the metabolic and inflammatory interactions underlying ORA. To our knowledge, this is the first study in a Croatian pediatric cohort to demonstrate this combined biomarker profile.

Overall, our findings support the concept that the obesity–asthma phenotype is multidimensional, characterized by complex interactions between metabolic dysregulation and airway inflammation. The coexistence of reduced adiponectin and elevated FeNO in obese asthmatic children, suggests that T2 and non-T2 mechanisms may operate simultaneously, varying between individuals and potentially influenced by treatment. The absence of significant differences in lung function, the lack of consistent associations between vitamin D status and inflammatory markers, and the potential confounding effects of ICS therapy further highlight the complexity of this phenotype. Therefore, while our results point toward a multifactorial relationship between obesity, vitamin D status, and asthma-related inflammation, they should be interpreted with caution. Future longitudinal and interventional studies are needed to clarify causal pathways and evaluate whether targeting metabolic factors, including adipokines and vitamin D status, may offer novel strategies for improving asthma outcomes in obese pediatric populations.

The increasing global prevalence of both childhood obesity and asthma highlights the importance of developing targeted prevention and management strategies. Lifestyle interventions, including weight reduction and increased physical activity, have been shown to improve asthma-related outcomes [75,76]. However, further research is required to determine their impact on asthma severity in children. Differentiating specific phenotypes and conducting larger population studies focusing on biomarkers such as adiponectin and vitamin D could improve personalized interventions and outcomes for pediatric patients with both conditions.

### Limitations of the Study

This study has several limitations that should be acknowledged. First, the relatively small cohort size may have resulted in statistical trends (e.g., *p* = 0.065), failing to reach significance due to low statistical power, particularly for subgroup analyses. Recruitment involved younger children, and sample collection occurred during the pandemic, which may have further constrained enrollment. In addition, the single-center design and recruitment from a geographically restricted population in Croatia resulted in a homogeneous cohort of Caucasian participants. As ethnic differences were not represented, the findings may not be generalizable to more diverse populations or different geographic settings. Larger, multicentric studies are needed to validate the observed trends and establish stronger associations between obesity, asthma, and inflammatory biomarkers.

Due to the retrospective nature of the study, only percent-predicted spirometry values were available in the dataset, and absolute values (FEV1, FVC, FEV1/FVC) required for z-score calculation were not recorded. Therefore, recalculation into GLI-2012 z-scores was unfortunately not feasible. Inclusion of z-scores would strengthen future prospective studies and enable cross-study comparability.

Although inhaled corticosteroid (ICS) treatment was included as a clinical variable, due to large data variability (different doses and classes of treatment within a relatively small sample size), ICS treatment was grouped as a binary variable (yes/no), not including data on dosage, duration, or adherence. As a result, analyses were restricted to comparing the proportion of children receiving any ICS treatment across groups. Consequently, the dose–response relationship or the impact of treatment intensity on FeNO levels could not be assessed.

Seasonal variability in 25-OH vitamin D3 levels represents another limitation. Blood samples were collected throughout the year without balanced distribution across the seasons, potentially confounding 25-OH vitamin D3 status measurements. Future studies should standardize sampling to specific seasons or include repeated seasonal measurements for each participant.

Although BMI was compared between the groups, it does not account for differences in body fat distribution. Future research should incorporate more detailed body composition assessments, such as measurements of visceral adiposity and lean mass, to better elucidate the role of fat distribution in asthma-related inflammation. Finally, additional potential confounders, including dietary intake, physical activity, and environmental exposures were not evaluated. These factors may influence both vitamin D levels and eosinophilic inflammation. Including such variables in future research would help clarify the complex interactions among lifestyle factors, obesity, and asthma phenotypes.

## 5. Conclusions

This study provides additional insight into the inflammatory profile associated with the obesity–asthma phenotype in children. Specifically, the observed reduction in adiponectin levels and the increase in FeNO suggest that T2 and non-T2 inflammatory mechanisms may coexist in obesity-related asthma rather than being a uniform phenotype and emphasize the influence of systemic metabolic factors on airway inflammation. In contrast, vitamin D levels did not show consistent differences between groups, and the findings may have been influenced by seasonal variability rather than reflecting true biological variation.

The novelty of this study lies in the integrated assessment of FeNO, adiponectin, and vitamin D status in a pediatric population, providing additional insight into the multidimensional nature of obesity-related asthma. However, the potential confounding effects of inhaled corticosteroid therapy and the lack of detailed treatment and control data warrant cautious interpretation of the results.

Overall, these results partially address the study objectives by supporting a role for adiponectin in the inflammatory mechanisms linking obesity and asthma while highlighting the need for further research to clarify the contribution of vitamin D. Future well-designed studies are required to better define whether targeting metabolic pathways—such as adipokine imbalance and vitamin D deficiency—may contribute to more personalized and effective management strategies in obese children with asthma.

## Figures and Tables

**Table 1:** Demographic characteristics with BMI values across the groups.

Parameter	Controls	Obesity	Asthma	Asthma + Obesity
Age (Mean ± SD)	10.0 ± 3.8	11.0 ± 3.4	11.1 ± 3.4	12.0 ± 2.8
Male (N)	15	17	16	17
Female (N)	15	13	14	13
BMI z-score, (SDS)	0.1 ± 0.9	2.1 ± 0.4	0.1 ± 1.0	2.0 ± 0.3

Abbreviations: BMI—body mass index; SD—standard deviation; N—number; z-score—standard score, SDS—standard deviation score.

**Table 2:** ICS therapy in asthmatic patients.

ICS Therapy	Asthma + Obesity *n* (%)	Asthma *n* (%)	*p*
Yes	9 (30%)	21 (70%)	0.002
No	21 (70%)	9 (30%)	

Abbreviations: ICS—inhaled corticosteroids.

**Table 3:** Lung function—spirometry results.

Parameter	Controls	Obesity	Asthma	Asthma + Obesity
FEV1 (%) predicted, Median (IQR)	94.0 (90.8–106.3)	102.5 (98.0–108.8)	98.0 (91.0–114.5)	102.0 (90.5–109.5)
FVC (%) predicted, Median (IQR)	85.0 (80.8–93.0)	93.5 (84.8–104.3)	96.5 (84.8–104.8)	94.0 (88.0–106.0)
FEV1/FVC predicted, Median (IQR)	114.8 (110.6–117.9)	107.4 (103.7–116.9)	104.9 (97.3–113.4)	104.5 (100.0–108.9)

Abbreviations: FEV1—Forced expiratory volume in the first second; FVC—Forced vital capacity; IQR—Interquartile Range.

**Table 4:** Serum 25-OH vitamin D levels and 25-OH vitamin D3 status by study group.

Parameter	Controls *n* (%)	Obesity *n* (%)	Asthma *n* (%)	Asthma + Obesity *n* (%)
25-OH Vitamin D3 (µg/L), Median (IQR)	23.9 (17.2– 34.0)	19.5 (10.0– 26.1)	20.2 (16.0– 27.2)	23.4 (17.7–28.9)
**25-OH Vitamin D3 status**				
Deficiency (<10 µg/L)	2 (7%)	7 (23%)	2 (7%)	2 (7%)
Insufficiency (10–19.9 µg/L)	10 (33%)	9 (30%)	11 (37%)	10 (33%)
Sufficient (≥20 µg/L)	18 (60%)	14 (47%)	17 (57%)	18 (60%)
Low vitamin D (<20 µg/L)	12 (40%)	16 (53%)	13 (43%)	12 (40%)

**Table 5:** Adiponectin levels in children with low vitamin D across study groups.

Group	Adiponectin (ng/mL), Median	Interquartile Range (IQR)	Controls	Obesity	Asthma	Asthma + Obesity
			*p* Value			
Controls	7.2	4.2–8.1	—	0.302	0.650	0.014 *
Obesity	4.3	2.9–8.1	0.302	—	0.503	0.443
Asthma	6.6	3.6–13.1	0.650	0.503	—	0.066
Asthma + Obesity	3.7	3.1–5.1	0.014 *	0.443	0.066	—

Mann–Whitney test, * *p* < 0.05.

## Data Availability

The data presented in this study are available on request from the corresponding author under specific conditions. The data are not publicly available due to ethical restrictions (sensitive data).

## Supplementary Materials

The following supporting information can be downloaded at: https://www.mdpi.com/article/10.3390/children13040514/s1, Figure S1 Ethics Committee approvals for the KK.01.1.1.07.0075 study, original in Croatian; Figure S2: Ethics Committee approvals for the KK.01.1.1.07.0075 study, translation to English; Figure S3. Ethics committee approval for the EDIAQI study, original in Croatian. Horizon Europe EDIAQI (Evidence driven indoor air quality improvement, grant agreement number 101057497); Figure S4. Ethics committee approvals for the EDIAQI study, translation to English. Horizon Europe EDIAQI (Evidence driven indoor air quality improvement, grant agreement number: 101057497); Figure S5. Ethics committee (at Srebrnjak Children’s Hospital) approval for the doctoral research of Jelena Knežević (Jelena Živković) entitled “Deficiency of vitamin D in obesity and asthma in the pediatric and adolescent population—potential modulatory factors”, original in Croatian; Figure S6. Ethics committee (at Srebrnjak Children’s Hospital) approval for the doctoral research of Jelena Knežević (Jelena Živković) entitled “Deficiency of vitamin D in obesity and asthma in the pediatric and adolescent population—potential modulatory factors”, translation to English; Figure S7. Ethics committee (of the School of Medicine, University of Zagreb, Croatia) approval for the doctoral research of Jelena Knežević (Jelena Živković) entitled “Deficiency of vitamin D in obesity and asthma in the pediatric and adolescent population—potential modulatory factors”, original in Croatian; Figure S8. Ethics committee (of the School of Medicine, University of Zagreb, Croatia) approval for the doctoral research of Jelena Knežević (Jelena Živković) entitled “Deficiency of vitamin D in obesity and asthma in the pediatric and adolescent population—potential modulatory factors”, translation to English.

## Abbreviations

The following abbreviations are used in this manuscript:

SCH	Srebrnjak Children’s Hospital
BMI	Body mass index
FeNO	Fraction of Exhaled Nitric Oxide
FEV1	Forced Expiratory Volume in 1 s
FVC	Forced Vital Capacity
ORA	Obesity-related asthma

## Appendix A

**Table A1:** FeNO levels and pairwise comparisons between study groups.

Group	FeNO (ppb), Median	Interquartile Range (IQR)	Controls	Obesity	Asthma	Asthma + Obesity
			*p* Value			
Controls	12.0	9.0–15.5	—	0.702	0.152	0.040 *
Obesity	10.0	5.5–19.0	0.702	—	0.038 *	0.021 *
Asthma	13.5	10.0–25.8	0.152	0.038 *	—	0.783
Asthma + Obesity	18.0	9.8–32.3	0.040 *	0.021 *	0.783	—

Abbreviations: FeNO—Fractional Exhaled Nitric Oxide; ppb—parts per billion. Mann–Whitney test, * *p* < 0.05.

**Table A2:** Adiponectin levels and pairwise comparisons between study groups.

Group	Adiponectin (ng/mL), Median	Interquartile Range (IQR)	Controls	Obesity	Asthma	Asthma + Obesity
			*p* Value			
Controls	7.1	4.2–9.3	—	0.100	0.792	0.024 *
Obesity	5.1	2.9–7.8	0.100	—	0.188	0.662
Asthma	6.5	3.7–12.9	0.792	0.188	—	0.065
Asthma + Obesity	4.4	3.3–7.1	0.024 *	0.662	0.065	—

Mann–Whitney test, * *p* < 0.05.
