# Supplementary material for: An Observational Study of the Role of Adiponectin and Vitamin D in Pediatric Asthma and Obesity
Source: Children (Basel). 2026 Apr 7;13(4):514. doi: 10.3390/children13040514 (PMC13114742; doi:10.3390/children13040514)
Supplement: Supplementary file 1 [file children-13-00514-s001.zip › children-4193387-supplementary.pdf]

SUPPLEMETARY MATERIALS

The role of adiponectin and vitamin D in childhood asthma and obesity in children

Figure S1 Ethics Committee approvals for the KK.01.1.1.07.0075 study, original in Croatian.

SREBRNJAK

Dječja bolnica Srebrnjak
Srebrnjak 100, Zagreb
Tel: 01 6391 100
Zagreb, 14.03.2022.
KLASA: 100-02/22-01
Ur.broj: 04-301/1-22

Članovi Etičkog povjerenstva su na 13. elektronskoj sjednici održanoj dana 14.03.2022. godine, s pet (5) glasova ZA donijeli slijedeću

ODLUKU

Članak 1.

Odobrava se provedba istraživačko-razvojnog projekta pod nazivom *„Razvoj personaliziranog koncepta za redukciju prekomjerne i održavanje zdrave tjelesne mase u liječenju kroničnih bolesti djece i odraslih"*, voditelj projekta izv.prof.dr.sc. Mirjana Turkalj, dr.med.

Članak 2.

Ova odluka stupa na snagu danom donošenja.

Obrazloženje

Prof.dr.sc. Davor Plavec, dr.med. podnio je Etičkom povjerenstvu dana 09.03.2022. godine Zamolbu za odobrenje provedbe istraživačko-razvojnog projekta pod nazivom „Razvoj personaliziranog koncepta za redukciju prekomjerne i održavanje zdrave tjelesne mase u liječenju kroničnih bolesti djece i odraslih", KK.01.1.1.07.0075, financiranog iz Europskog fonda za regionalni razvoj, iz operativnog programa Konkurentnost i kohezija, Poziva jačanje kapaciteta za istraživanje, razvoj i inovacije. Voditelj projekta je izv.prof.dr.sc. Mirjana Turkalj, dr.med..
Članovi Etičkog povjerenstva su sa pet glasova ZA odobrili provedbu istraživanja.
Sukladno navedenom, odlučeno je kao u Izreci ove Odluke.

Predsjednica Etičkog povjerenstvo
doc.dr.sc. Helena Munivrana Škvorc, dr.med.

Doc. prim. dr. sc.
Helena Munivrana Škvorc, dr. med.
specijalist pedijatar
alergolog i klinički imunolog
9185765

Dostaviti:
1. Podnositelju zamolbe
2. Arhiva

QUALITY SYSTEM CERTIFICATION
DNV·GL
ISO 9001:2015

Figure S2: Ethics Committee approvals for the KK.01.1.1.07.0075 study- translation to English.

On 14th March 2022 at the 20th Ethics Committee session, with 5 votes PRO the members of the Ethics Committee have reached the following

DECISION

Article 1.

The implementation of the research project and the clinical study within the ***project entitled „Development of a personalized concept for the reduction of excess and maintenance of healthy body weight in the management of chronic diseases in children and adults“***, Principal Investigator: Prof. Mirjana Turkalj, MD, PhD has been APPROVED hereby.

Article 2.

This decision becomes final on the day it is reached.

Elaboration

On 9th March 2022 prof. Davor Plavec, MD, PhD has submitted the request for approval of the research project ERDF „Development of a personalized concept for the reduction of excess and maintenance of healthy body weight in the management of chronic diseases in children and adults“, funded by the European Regional and Development Fund, under the Operational Program Competitiveness and Cohesion, call for capacity building for research, development, and innovation (grant agreement number: KK.01.1.1.07.0075), along with relevant ethics related documentation. The Principal Investigator of the study is Prof. Mirjana Turkalj, MD, PhD.

According to this request, the members of the Ethics Committee of the Srebrnjak Children`s Hospital at the 13th Ethics Committee session held online on 14th March 2022 have found this study and the research project valid and ethically acceptable, thus reaching the afore mentioned Decision.

The President of the Ethics Committee of the SRebrnjak Children`s Hospital

Assoc. Prof. Prim. Helena Munivrana Škvorc, MD, PhD

Deliver to:

1. The applicant

2. Archives

Figure S3. Ethics committee approval for the EDIAQI study, original in Croatian. Horizon Europe EDIAQI (Evidence driven indoor air quality improvement, grant agreement number 101057497).

SREBRNJAK

Dječja bolnica Srebrnjak
Srebrnjak 100, Zagreb
Tel: 01 6391 100
Zagreb, 01.02.2023.
KLASA: 100-02/23-01
Ur.broj: 04-100/3-23

Članovi Etičkog povjerenstva su na 20. sjednici održanoj dana 01.02.2023. godine, sa pet glasova ZA donijeli slijedeću

ODLUKU

Članak 1.

Odobrava se provedba znanstveno-istraživačkog projekta pod nazivom ***EU H2020 EDIAQI („Evidence Driven Indoor Air Quality Improvement“),*** voditelj projekta prof.dr.sc. Mirjana Turkalj, dr.med.

Članak 2.

Ova odluka stupa na snagu danom donošenja.

Obrazloženje

Dr.sc. Ivana Banić, mag.mol.biol. podnijela je Etičkom povjerenstvu Zamolbu za odobrenje provedbe znanstveno-istraživačkog projekta pod nazivom EU H2020 EDIAQI („Evidence Driven Indoor Air Quality Improvement“), financiranog sredstvima EU za istraživanje i inovacije u sklopu okvirnog programa Obzor 2020 (Horizon 2020), Grant agreement ID: 101057497. Voditelj projekta je prof.dr.sc. Mirjana Turkalj, dr.med..
Sukladno podnesenoj Zamolbi, članovi povjerenstva su na 20. elektronskoj sjednici dana 01.02.2023. ocijenili provedbu znanstvenog istraživanja etički prihvatljivim, te je donesena Odluka kao u Izreci.

Predsjednica Etičkog povjerenstvo
prim.doc.dr.sc. Helena Munivrana Škvorc, dr.med.

Doc. prim. dr. sc. Helena Munivrana Škvorc, dr. med.
specijalist pedijatar
alergolog i klinički imunolog
185765

Dostaviti:
1. Podnositelju zamolbe
2. Arhiva

Figure S4. Ethics committee approvals for the EDIAQI study- translation to English. Horizon Europe EDIAQI (Evidence driven indoor air quality improvement, grant agreement number: 101057497).

On 1st February 2023 at the 20th Ethics Committee session, with 5 votes PRO the members of the Ethics Committee have reached the following

DECISION

Article 1.

The implementation of the research project and the clinical study within the ***EU Horizon 2020 EDIAQI ("Evidence Driven indoor Air Quality Improvement")***, Principal Investigator: Prof. Mirjana Turkalj, MD, PhD has been APPROVED hereby.

Article 2.

This decision becomes final on the day it is reached.

Elaboration

Ivana Banić, PhD, MSc in Mol Biol has submitted the request for approval of the research project EU Horizon 2020 ("Evidence Driven Indoor Air Quality Improvement"), funded by the EU Horizon framework programme for research and innovation, Grant agreement ID: 101057497, along with relevant ethics related documentation. The Principal Investigator of the study is Prof. Mirjana Turkalj, MD, PhD.

According to this request, the members of the Ethics Committee of the Srebrnjak Children`s Hospital at the 20th Ethics Committee session held online on 1st February 2023 have found this study and the research project valid and ethically acceptable, thus reaching the afore mentioned Decision.

The President of the Ethics Committee of the SRebrnjak Children`s Hospital

Assoc. Prof. Prim. Helena Munivrana Škvorc, MD, PhD

Deliver to:

1. The applicant

2. Archives

Figure S5. Ethics committee (at the Srebrnjak Children`s Hospital) approval for the doctoral research of Jelena Knežević (Jelena Živković) entitled "Deficiency of vitamin D in obesity and asthma in the pediatric and adolescent population-potential modulatory factors", original in Croatian.

SREBRNJAK

Dječja bolnica Srebrnjak
Srebrnjak 100, Zagreb
Tel: 01 6391 100
Zagreb,16.4.2020.
Broj: 2/2020

Etičko povjerenstvo u sastavu:
Prof. Mirjana Turkalj, dr.med.-predsjednica
Prof. Željko Tanjić, zamjenik predsjednice
Prof. Davor Plavec, dr.med.-član
Prim. Lana Bukovac Tambić, dr.med.-član
Doc.dr.sc. Damir Erceg, dr.med.-član

Jelena Živković, mag.biol.exp.
Dječja bolnica Srebrnjak
Srebrnjak 100
10000 Zagreb

MIŠLJENJE ETIČKOG POVJERENSTVA DJEČJE BOLNICE SREBRNJAK

Na temelju zahtjeva i dostavljene dokumentacije pristupnice Jelene Živković, mag.biol.exp., Etičko povjerenstvo Dječje bolnice Srebrnjak na redovnoj održanoj sjednici 21.2.2020., razmotrilo je zamolbu pristupnice i pristiglu dokumentaciju te donijelo:

ODLUKU

O odobrenju provođenja istraživanja za potrebe izrade doktorske disertacije pristupnice Jelene Živković, mag.biol.exp., pod naslovom: **"Nedostatak vitamina D kod pretilosti i astme u pedijatrijskoj i adolescentnoj populaciji – potencijalni modulatorni čimbenici"**

Mentor doktorskog rada pristupnice je Izv. prof. dr. sc. Mirjana Turkalj, dr. med., Dječja bolnica Srebrnjak, Medicinski fakultet Osijek i Hrvatsko katoličko sveučilište.

Odluka se temelji na slijedećim poslanim dokumentima:
1. Zamolba za dobivanje mišljenja Etičkog povjerenstva
2. Obrazloženje teme doktorske disertacije
3. Obavijest za ispitanika o sudjelovanju u istraživanju
4. Informirani pristanak
5. Informirani pristanak za adolescente
6. Informirani pristanak za pedijatrijsku populaciju
7. Informirani pristanak za genetiku

Zamjenik predsjednice Etičkog povjerenstva
Prof. Željko Tanjić

Željko Tanjić

QUALITY SYSTEM CERTIFICATION DNV-GL ISO 9001:2015

Figure S6. Ethics committee (at the Srebrnjak Children`s Hospital) approval for the doctoral research of Jelena Knežević (Jelena Živković) entitled "Deficiency of vitamin D in obesity and asthma in the pediatric and adolescent population-potential modulatory factors", translation to English.

Ethics Committee members:

Prof. Mirjana Turkalj, MD, PhD- Ethics Committee President

Prof. Željko Tanjić- Ethics Committee Vice-president

Prof. Davor Plavec, MD, PhD- member

Prim. Lana Bukovac, MD- member

Assoc. Prof. Damir Erceg, MD, PhD- member

OPINION OF THE ETHICS COMMITTEE OF SREBRNJAK CHILDREN`S HOSPITAL

Based on the requested and submitted documentation of the applicant Jelena Živković, MSc in Experimental Biology, the Ethics Committee of the Srebrnjak Children`s Hospital at its regular session held on 21st February 2020 has reviewed the applicant`s request and relevant documentation and has reched the following

DECISION

The implementation of the research within the doctoral research and thesis pof the applicant Jelena Živković, MSc in Exp. Biol. entitled **„Deficiency of vitamin D in obesity and asthma in the pediatric and adolescent population – potential modulatory factors".**

The doctoral thesis mentor is: Prof. Mirjana Turkalj, MD, PhD, Srebrnjak Children`s Hospital, Faculty of Medicine, J.J. Strossmayer University of Osijek, Catholic University of Croatia.

The Decision is based on the following documentation:

1. Request for Ethics Committee approval
2. Elaboration of the doctoral thesis topic
3. Informed consent form- written information reagrding the study
4. Informed consent form
5. Informed consent form for adolescents
6. Informed consent for for the pediatric population
7. Informed consent form for genetic research

The Vice-president of the Ethics Committee of the Srebrnjak Children`s Hospital

Prof. Željko Tanjić

____

Figure S7. Ethics committee (of the School of Medicine, University of Zagreb) approval for the doctoral research of Jelena Knežević (Jelena Živković) entitled "Deficiency of vitamin D in obesity and asthma in the pediatric and adolescent population- potential modulatory factors", original in Croatian.

Sveučilište u Zagrebu
Medicinski fakultet

A Šalata 3
10000 Zagreb

T +385 1 45 66 777
F +385 1 49 20 053

E mf@mef.hr
W www.mef.unizg.hr

ETIČKO POVJERENSTVO

Ur. Broj: 380-59-10106-20-111/87
Klasa: 641-01/20-02/01

Zagreb, 25.6.2020.

Jelena Živković, mag.exp.biol.
Dječja bolnica Srebrnjak
Srebrnjak 100
10 000 Zagreb

Mišljenje Etičkog povjerenstva

Etičko povjerenstvo MEDICINSKOG FAKULTETA u Zagrebu razmotrilo je načela etičnosti istraživanja u sklopu izrade disertacije **Jelene Živković, mag.exp.biol.** pod naslovom:

„Nedostatak vitamina D kod pretilosti i astme u pedijatrijskoj i adolescentnoj populaciji – potencijalni modulatorni čimbenici"

i zaključilo da je prikazano istraživanje etički prihvatljivo.

Prof. dr. sc. Zdravka Poljaković
Predsjednica Etičkog povjerenstva

Dostavljeno: 1. Podnositeljici zahtjeva
2. Arhiva Povjerenstva

Figure S8. Ethics committee (of the School of Medicine, University of Zagreb) approval for the doctoral research of Jelena Knežević (Jelena Živković) entitled "Deficiency of vitamin D in obesity and asthma in the pediatric and adolescent population- potential modulatory factors", translation to English.

University of Zagreb

School of Medicine **ETHICS COMMITTEE**

Šalata 3

10000 Zagreb

Ref. No.: 380-59-10106-20-111/87

Class: 641-01/20-02/01

Zagreb, 25th June 2020 Jelena Živković, MSc in Exp. Biol.

Ethics Committee opinion

The Ethics Committee of the SCHOOL OF MEDICINE, University of Zagreb has reviewed the ethics principles of the research within the doctoral thesis of **Jelena Živković, MSc in Exp. Biol.** entitled:

„Deficiency of vitamin D in obesity and asthma in the pediatric and adolescent population- potential modulatory factors"

and has reached the conclusion that the afore mentioned research is ethically acceptable.

Prof. Zdravka Poljaković, PhD, MD

Ethics Committee Chair

Deliver to:

1. The Applicant
2. 2. Ethics Committee archives
